# Uncovering biodegradability and biocompatibility of betaine-based deep eutectic systems

**DOI:** 10.1007/s11356-022-25000-6

**Published:** 2023-01-06

**Authors:** Inês João Ferreira, Alexandre Paiva, Mário Diniz, Ana Rita Duarte

**Affiliations:** 1 LAQV-REQUIMTE, Department of Chemistry, School of Science and Technolog, NOVA University Lisbon, 2829-516 Caparica, Portugal; 2 Associate Laboratory i4HB - Institute for Health and Bioeconomy, School of Science and Technology, NOVA University Lisbon, 2819-516 Caparica, Portugal; 3 UCIBIO – Applied Molecular Biosciences Unit, Department of Chemistry / Department of Life Sciences, School of Science and Technology, NOVA University Lisbon, 2819-516 Caparica, Portugal

**Keywords:** Deep eutectic systems, Green solvents, Environmental toxicity, Cytotoxicity

## Abstract

Deep eutectic systems (DES) have shown increasing popularity in last decade; however, the number of studies on the potential toxicity towards living organisms remains scarce. These studies are of the utmost importance to infer on the claimed non-toxicity and biocompatibility of DES. Most articles published, at this moment, only evaluate the toxicity towards a cell model or in different strains of bacteria. For this purpose, in this work, the effect of two DES (betaine:sorbitol:water 1:1:3 and betaine:glycerol 1:2) and their individual components were evaluated at different concentrations after administered via intraperitoneal injection in zebrafish (*Danio rerio*). The total antioxidant capacity, lipoperoxidation, and the activity of various enzymes that work in different antioxidant pathways (superoxide dismutase, glutathione peroxidase, catalase, and glutathione S-transferase) were assessed. The results show no significant toxicity within the tested concentrations: up to 5000 µM and 3000 µM, for the assays using the system betaine:sorbitol:water 1:1:3 and for betaine:glycerol 1:2, respectively. The toxicity of individual components was studied up to 1000 µM. Based on the encouraging results that have been obtained, it is safe to conclude that these two deep eutectic systems can be used as the new class of environmentally friendly solvents.

**Graphical Abstract:**

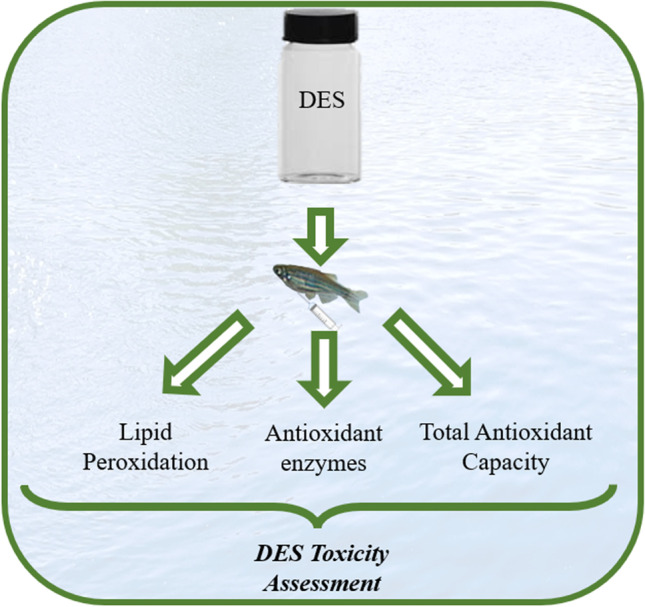

**Supplementary Information:**

The online version contains supplementary material available at 10.1007/s11356-022-25000-6.

## Introduction

Deep eutectic systems are included in the class of neoteric solvents (Gotor-Fernández and Paul 2019). Neoteric solvents appear as a viable alternative to toxic and harmful solvents in respect to environmental questions, which is one of the key topics under development in Green Chemistry. This class has gained supporters due to the diverse applications in different industries. However, the neoteric solvents’ overall effect on the environment is still up for debate as more studies are needed to reach definitive conclusions (Cañadas et al. 2020).

DES were first described by Abbot and co-workers (Abbott et al. 2003, 2004), based on the observation that a mixture of urea and an ammonium salt, in a given molar ratio, would form a liquid, due to the melting point depression of the system, as a result of the interactions between the two components. DES can, hence, be described as a mixture of two or more components, at least one of which is a hydrogen bond donor (HBD) and the other a hydrogen bond acceptor (HBA), which, when combined in a certain molar ratio, results in a marked decrease in the melting point of the initial components (Ferreira et al. 2022; Liu et al. 2015; Mbous et al. 2017; Mouden et al. 2017; Smith et al. 2014; Tang et al. 2015). Another extremely relevant physical characteristic of DES is the viscosity, which is higher in these systems than in common organic solvents and water. This is due to extensive hydrogen bonding network, and also Van der Waals interactions (Skulcova et al. 2019). Deep eutectic systems are easy to prepare; may start from cheap, natural, and renewable raw materials; and after preparation do not require further purification steps (Hayyan et al. 2013; Jhong et al. 2009; Singh et al. 2012). Currently, DES have several applications as solvents (Clarke et al. 2018; Radošević et al. 2018; Stott et al. 1998), in chemical processes and in extraction (Duan et al. 2016; Jiang et al. 2017), catalysis processes (Gutiérrez et al. 2010; Khodaverdian et al. 2018), separations (Delgado-Rangel et al. 2020; Xu et al. 2019), organic synthesis (Brandão et al. 2013; Saleem Khan et al. 2022), electrodeposition (Alcanfor et al. 2017; Ghosh and Roy 2015), nanomaterials (Karimi and Eshraghi 2017; Renjith and Lakshminarayanan 2015), and in biomedical (Jian et al. 2020) and pharmaceutical (Al-Akayleh et al. 2019; Angsantikul et al. 2021; Stott et al. 1998) industries.

In fact, there is more than one subclass of DES, among them are the natural deep eutectic systems (NADES), which were first introduced in 2011 by Choi et al. 2011 as a mixture of natural compounds, such as, alcohols, choline chloride, amines, acids, amides, and sugars (Paiva et al. 2014; Radošević et al. 2018). Another important characteristic of NADES is their biological or physiologic function in nature (Vanda et al. 2018).

NADES can be applied in different industries (cosmetic, pharmaceutical, and food) and also in enzymatic processes. In the pharmaceutical industry, the main advantages of NADES are their capacity to dissolve macromolecules and pharmaceuticals ingredients (Vanda et al. 2018) which would otherwise be insoluble in conventional media or even to enhance the bioavailability of some drugs (Benlebna et al. 2018). However, the toxicity of NADES must be evaluated, not only if NADES are to be used in human consumption, but also in which concerns environmental contamination (Murador et al. 2019).

For example, in 2021, Jesus and co-workers determined the in vitro toxicity against the cell line L929 of two different DES based on betaine and glycerol (betaine:glycerol:sucrose:water (2:3:1:5) and betaine:glycerol:trehalose:water (2:3:1:5)) (Jesus et al. 2021). It was observed that sucrose is slightly more toxic than trehalose, with an IC50 of 1.7 M and 2 M respectively. However, when cells were incubated with 5% of NADES in complete media, the viability of L929 cells was two times higher than for dimethyl sulfoxide (Me_2_SO). When NADES concentration increases to 10%, the difference in viability is not significant, but it continues to be higher than the viability in Me_2_SO. Another study assessed the in vitro toxicity of betaine-based DES using a human intestinal cell model, Caco-2 (Rodrigues et al. 2021). The authors tested three different DES combinations (betaine:glycerol (1:2), betaine:propylene glycol (1:3), and betaine:ethylene glycol (1:3)) with different ratios of water. In summary, DES were less toxic for the type of cells tested (Caco-2) than betaine, however, more toxic than isolated polyols with Bet:EG (1:3) being less toxic than Bet:Gly (1:2) and this one less toxic than Bet:PG (1:3). The same authors also determined the minimum inhibitory concentration (MIC) in Gram-negative (*E. Coli*) and Gram-positive (*S. aureus*) bacteria of these DES. The results indicated that the increase in water content is responsible for an increase in MIC, being Bet:EG (1:3) the DES with lower MIC and Bet:PG (1:3) the DES showing the highest value. The authors also tested the leaves harvested from wheat seedlings exposed to DES, and in some cases they observed a decrease in the activity of different antioxidant enzymes (e.g., SOD, CAT, and guaiacol). Benlebna et al. (2018) evaluated the in vivo toxicity of the Bet:Gly (2:1) NADES extract of green Arabic coffee beans. The NADES extract, rich in phenolic compounds, resulted in a mortality of one third of the mice tested. This was associated with a hepatomegaly, dietary restriction, weight loss, excessive water consumption, plasma oxidative stress, adipose tissue loss, and increased blood lipid levels. The use of NADES and DES is anticipated to rise in the upcoming years. Within this context, this work can help to comprehend the effects of these systems on the environment, specifically aquatic biota, since these compounds will eventually reach aquatic ecosystems via wastewater discharges. Additionally, the findings might also promote the understanding of how other species, including humans, might be affected and encourage industry to use them safely. Therefore, the primary goals of this study are to determine the toxicity after intraperitoneal injection in zebrafish (*Danio rerio*), a standardized animal model that is frequently used in toxicology and biomedicine research, of two different betaine-based DES, betaine:glycerol (Bet:Gly) and betaine:sorbitol:water (Bet:Sor:W) in a 1:2 and 1:1:3 molar ratio, respectively. Therefore, lipid peroxidation, total antioxidant capacity and the activity of some oxidative stress enzymes (superoxide dismutase, glutathione-S-transferase, catalase) were measured. The present work provides helpful information on betaine-based deep eutectic systems effects in freshwater fish species.

## Experimental section

### Preparation of DES

Betaine anhydrous (Bet) (CAS # 107–43-7, TCI, purity: ≥ 97%, USA), glycerol (Gly) (CAS # 56–81-5, Scharlau, purity: 99,5, Spain), and D-sorbitol (Sor) (reference: 57,876–0500, Sigma-Aldrich, Germany) were used to prepare the DES. Briefly, betaine was gently mixed with glycerol at a molar ratio of 1:2 and constantly stirred at 70 °C, until a clear liquid solution forms. On the other hand, betaine was also gently mixed with sorbitol and distilled water in a ratio of 1:1:3 and subsequently stirred at 70 °C, until a clear liquid solution forms.

### Bioassays

#### Exposure of zebrafish to DES via intraperitoneal injection

Zebrafish (*Danio rerio*) were purchased from the commercial supplier Aquaplante (Portugal) and after they were acclimatized for as a minimum of 48 h in a lab setting. A closed-circuit system with filtered, dechlorinated tap water was used to maintain the fish, at a temperature of 20 ± 1 °C, at pH 7.2 ± 0.1, and with constant aeration (> 6 mg O_2_ L^−1^). The photoperiod was 12 h light and 12 h darkness.

##### Zebrafish exposure to Bet:Gly (1:2), Bet and Gly

Since sex could not be determined by external observation, adult fish (*n* = 98; 0.168 ± 0.050 g) of both genders were divided into four different groups randomly and injected each one with 10 µL of Hank’s solution (“Common Buffers, Media, and Stock Solutions,” 2000), DES (Bet:Gly (1:2)), betaine, or glycerol. Dilutions with Hank’s solution and the saline solution were carried out (“Common Buffers, Media, and Stock Solutions,” 2000). The last three groups were subdivided in 3 different concentrations: 250 µM, 500 µM, and 1000 µM of Gly or Bet and 750 µM, 1500 µM, and 3000 µM of Bet:Gly (1:2). The distribution of animals by group is showed in Table 1.

**Table 1:** Number of animals for group

Compound	Concentration	Number of animals
Control		8
Bet: Gly (1:2)	750 µM	10
	1500 µM	10
	3000 µM	10
Bet	250 µM	10
	500 µM	10
	1000 µM	10
Gly	250 µM	10
	500 µM	10
	1000 µM	10

##### Zebrafish exposure to Bet:Sor:W (1:1:3)

After becoming acclimated, adult zebrafish (*n* = 38; 0.181 ± 0.052 g) of both genders have been randomly placed into four different aquariums and injected each one with 10 µL of DES (Bet:Sor:W (1:1:3)) or Hank’s solution (“Common Buffers, Media, and Stock Solutions,” 2000) by intraperitoneal injection in different concentrations (0 µM; 1250 µM; 2500 µM; and 5000 µM). Dilutions with Hank’s solution (“Common Buffers, Media, and Stock Solutions,” 2000) were carried out, and the same was used to inject the controls. The distribution of animals by concentration is showed in Table 2.

**Table 2:** Number of animals by concentration of Bet:Sor:W (1:1:3) administered

Compound	Concentration	Number of animals
Bet:Sor:W (1:1:3)	0 µM	8
	1250 µM	10
	2500 µM	10
	5000 µM	10

The experiments were carried out for 96 h, and the fish were fed every day, ad libitum, with store-bought flakes (Tetra brand, USA). The pH and the temperature of the aquarium were monitored, and total mortality was recorded. After the experiment period of time fish were euthanized through quick freezing at − 45 °C. Animals were then weighed and reserved at − 45 °C until processing.

### Biochemical assays

#### Samples treatment

Using a tissue homogenizer (Tissue Master 125, USA), entire fish were homogenized in 3 mL of cold PBS at pH 7.4 ± 0.2. Centrifuging tissue homogenates at 4 °C for 10 min at 15,0000 × g (VWR, by Hitachi Koki Co., Ltd), stored at − 45 °C, and analyzed in the following days. The total mass of cytosolic proteins as determined by the Bradford method (Bradford 1976) was used to normalize all biochemical assays results.

#### Glutathione S-transferase (GST)

The procedure originally described by Habig et al. (1974) was used to determine the total GST activity (EC 2.5.1.18) and modified for 96-well microplates. This procedure was based on the creation of a conjugate by the reaction of reduced glutathione and 1-chloro-2,4-dinitrobenzene (cDNB), which can be followed at 340 nm absorbance. Briefly, 20 µL of samples from each well were added to 180 µL of the substrate solution, constituted by a mixture of PBS (“Common Buffers, Media, and Stock Solutions,” 2000) with 200 mM reduced glutathione (Sigma-Aldrich, Germany) and 100 mM cDNB (Sigma-Aldrich, Germany). Using a microplate reader (Synergy HTX, BioTek, USA), the absorbance was recorded every minute for 10 min to measure the whole enzyme activity at 340 nm. A cDNB extinction coefficient (0.0053 µM^−1^ cm^−1^) was used to calculate the change in absorbance for each minute and the reaction rate at 340 nm. The results are expressed based on the total cytosolic protein concentration calculated for each sample (nmol.min^−1^ mg^−1^ total cytosolic protein).

##### Catalase (CAT)

CAT activity (EC 1.11.1.6) was determined according to a method developed by Johansson and Håkan Borg (1988) and adapted to a 96-well microplate. In each microplate well (Greiner Bio-one, Austria) was added 20 µL of formaldehyde or sample, 100 µL of buffer (potassium phosphate 100 mM, pH 7.0), and 30 µL of methanol (Scharlau, Spain). The reaction starts, after adding 20 µL of hydrogen peroxide in a concentration of 0.035 M (Sigma Aldrich, Germany) to the microplate. Afterwards, the microplate was shaken vigorously for 20 min. Following that, 30 µL of potassium hydroxide at a concentration of 10 M (Chem-Lab, Belgium) and 30 µL of purpald (in a concentration of 34.2 M in 0.5 M HCl) (Aldrich, Germany) were added and allowed to incubate for 10 min. In each microplate well, 10 µL of potassium periodate at a concentration of 65.2 mM (Chem-Lab, Belgium) was added, and the absorbance was measured, in a microplate reader, at 540 nm following five minutes of incubating in darkness. The samples’ formaldehyde levels were calculated using a calibration curve with a range of 0 to 75 M (Sigma-Aldrich, Germany). Based on formaldehyde equivalents, catalase activity was calculated. The results are represented in relation to the sample’s total cytosolic protein concentration (nmol.min^−1^ mg^−1^ total cytosolic protein).

##### Glutathione peroxidase (GPX)

Glutathione peroxidase activity (GP_x_) (EC 1.11.1.9) was measured using a 96-well microplate based on the method of Lawrence and Burk (1976) that was adapted. Succinctly, 20 μL of each sample, 120 μL of assay buffer (constituted by potassium phosphate buffer (Sigma-Aldrich, Germany) in a concentration of 50 mM at pH 7.4 and EDTA in a concentration of 5 mM (Riedel-Haen, Germany)), and 50 μL of the co-substrate mixture were applied to each well. Sodium azide (Sigma-Aldrich, Germany) in a concentration of 4 mM, nicotinamide adenine dinucleotide phosphate (NADPH, Roche, Germany) in a molar concentration of 1 mM, glutathione reductase (GSSG-reductase) in a concentration of 4 U/mL, and reduced glutathione in a concentration of 4 mM made up the co-substrate mixture.

A 20 μL of 15 mM hydroperoxide cumene (Sigma-Aldrich, Germany) was added to start the reaction, and absorbance at 340 nm was measured each minute for 6 min using a microplate reader. The extinction coefficient for β-NADPH (3.73 mM^−1^ cm^−1^) was used to calculate the reaction rate such as the decline in absorbance per minute (ΔA_340_). The results were expressed in relation to the total cytosolic protein concentration of the samples (nmol.min^−1^ mg^−1^ total cytosolic protein).

##### Superoxide dismutase (SOD)

Superoxide dismutase (SOD) activity (EC 1.15.1.1) was calculated following the NBT (nitroblue tetrazolium) method, previously described by Sun et al. (1988) and adapted to a 96-well microplate. In this technique, xanthine and xanthine-oxidase (XOD) react to produce superoxide radicals (•O_2_^−^), and NBT is reduced to formazan, which can be detected at 560 nm. Thus, the SOD activity measured in samples was determined as the percent inhibition (% inhibition) of the rate of NBT-diformazan formation and then converted to units per mg of total cytosolic protein. In each well, 200 μL of phosphate buffer in a concentration of 50 mM at pH 8.0, 10 μL of xanthine (Sigma-Aldrich, Germany) in a concentration of 3 mM, 10 μL of NBT (Sigma-Aldrich, Germany) in a concentration of 0.075 mM, and 10 μL of the sample were added. The reaction was initiated by adding 10 μL of XOD (Sigma-Aldrich, Germany). Using a plate reader, the absorbance at 536 nm was then recorded every two minutes for 26 min. Negative controls had every component except the sample, which produced a maximal increase in absorbance at the wavelength measured. The SOD results are expressed as units per mg of total cytosolic protein.

##### Total antioxidant capacity (TAC)

The Kambayashi et al. (2009) method was used to determine total antioxidant capacity (TAC). The sample (10 µL) was added to a 96-well microplate. Then, myoglobin (10 µL) (Sigma, Germany) at 90 µM and 150 µL of ABTS (2,2′-azino-bis-3-ethylbenzothiazoline-6-sulfonic acid) (Alfa Aesar, Germany) at a concentration of 600 µM were also added. In the end, 40 µL of hydrogen peroxide (Sigma-Aldrich, Germany) at a concentration of 500 µM begins the reaction. With a microplate reader, the absorbance was measured at 415 nm after 5 min of incubation. Trolox was used as the standard, and a calibration curve with a range of 0 to 0.33 mM was used to determine TAC. The results are presented in relation to the total cytosolic protein concentration of each sample (nmol.mg^−1^ total cytosolic protein).

##### Lipid peroxide assay (MDA content)

Lipid peroxide assay was modified from the TBARS protocol (thiobarbituric acid reactive substance) (Uchiyama and Mihara 1978). In a microtube, 45 μL of PBS (pH 7–7.4) was added to 5 μL of each sample. Each microtube was then filled with 12.5 μL of SDS (Sigma-Aldrich, Germany) at a concentration of 8.1% (w/v), 93.5 μL of trichloroacetic acid (Panreac, Spain) at a concentration of 20% (w/v), and 93.5 μL of thiobarbituric acid (Sigma-Aldrich, Germany) at a concentration of 1% (w/v). Each microtube was then filled with 50.5 μL of MQ-grade ultrapure water before being stirred in a vortex for 30 s. The microtubes’ lids were pierced with a needle, and after 10 min in boiling water, they were immediately put on ice for a short while to cool. Then, each microtube received 62.5 μL of MQ-grade ultrapure water. After that, the microtubes were mixed for a minute. Each well of a 96-well microplate received a duplicate 150 μL of each microtube, and each well’s absorbance was measured at 530 nm using a microplate reader. Malondialdehyde bis(dimethylacetal) (MDA) (Merck) was used as the standard to create a ten-point calibration curve (0–0.1 μM TBARS) to quantify lipid peroxides. The results are represented in relation to the total cytosolic protein concentration of the sample (pmol.mg^−1^ total cytosolic protein).

### Statistical analysis

Statistical comparisons were conducted using the one-way ANOVA or Kruskal–Wallis test hunted by Dunnett’s multiple comparisons test. Additionally, using the non-parametric Spearman rank *R* test, correlation analyses between the examined biomarkers were carried out.

## Results

The DES prepared in this work have been designed for the evaluation of their potential as enzyme stabilizing agents (Gajardo-Parra et al. 2022). Their use in a large industrial scale requires their full characterization not only in terms of their physico-chemical properties, but also their ecotoxicological profile. The physico-chemical characterization is presented in a previous paper (Gajardo-Parra et al. 2022), and hence here we focus mostly on their toxicity profile.

### Effects of system Bet:Gly (1:2) and respectively individual compounds injected intraperitoneally

#### Mortality rate

The mortality rate registered throughout the assay was not significant (Table 3).

**Table 3:** Percentage of death of *Danio Rerio* by concentration and compound (Bet:Gly (1:2), Betaine and Glycerol)

	**0 µM**	**250 µM**	**500 µM**	**750 µM**	**1000 µM**	**1500 µM**	**3000 µM**
Betaine:glycerol (1:2)	0%	––	––	0%	––	0%	0%
Betaine	0%	0%	10%	––	10%	––	––
Glycerol	0%	0%	20%	––	0%	––	––

#### Glutathione S-transferase

**Fig. 1:**
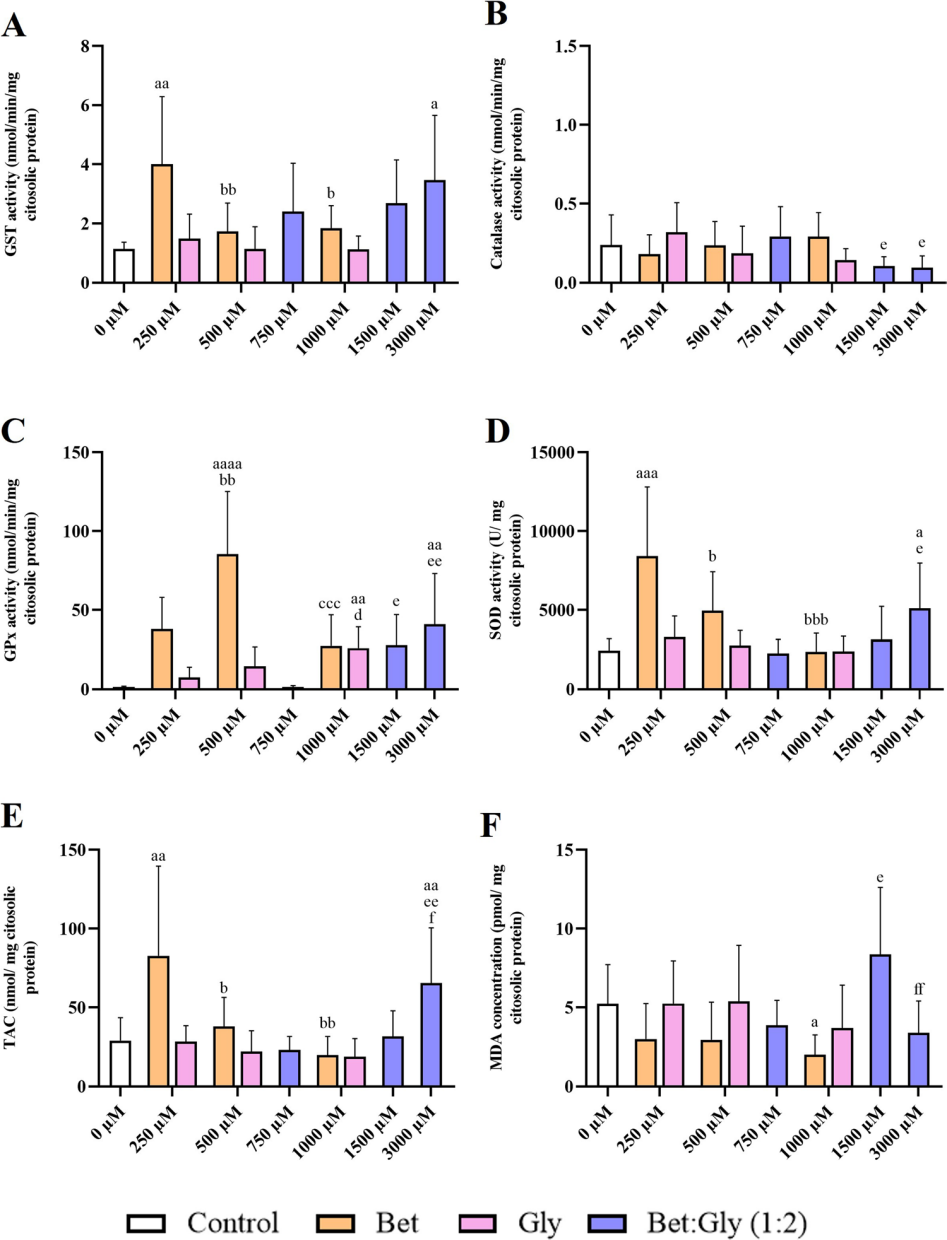
**A** GST activity, **B** CAT activity, **C** GPx activity, **D** SOD activity, **E** TAC, and **F** MDA concentration after *D. rerio* injection with different concentrations of Bet:Gly (1:2), betaine, and glycerol. All data was represented as mean ± s.d.. a, significant differences in comparison to control; b, significant differences compared with 250 µM betaine; c, significant differences compared with 500 µM of betaine; d, significant differences compared with 250 µM glycerol; e, significant differences compared with 750 µM of Bet:Gly (1:2); f, significant differences compared with 1500 µM of Bet:Gly (1:2). One letter corresponds to significant differences with *p* < 0.05, two repeated letters to *p* < 0.01, three repeated letters *p* < 0.001, and 4 repeated letters to *p* < 0.0001

The GST activities mean concentrations measured in *D. rerio* are presented in Fig. 1A. Regarding the activity of these enzyme in animals exposed to DES, a significant increase (*p* < 0.05) was observed comparing fish injected with saline solution with fish injected with 3000 µM DES (Bet:Gly (1:2)). In respect to GST activity in animals injected with betaine, there was a significant increase (*p* < 0.01) compared to fish injected with saline solution or with fish injected with 250 µM of betaine. A significant decrease (*p* < 0.01, *p* < 0.05) was also observed when comparing fish injected with 250 µM of betaine and those injected with 500 µM and 1000 µM, respectively.

#### Catalase

The CAT activity mean concentrations measured in *D. rerio* are presented in Fig. 1B. Regarding catalase activity in animals exposed to Bet:Gly (1:2), a significant decrease (*p* < 0.05) was observed comparing fish intraperitoneally injected with 750 µM of DES (Bet:Gly (1:2)) and fish intraperitoneally injected with 1500 µM and 3000 µM of DES.

#### Glutathione peroxidase activity

Figure 1C presents the mean concentration of GP_x_ activity determined in *D. rerio*. Regarding GP_x_ activity in animals exposed to Bet:Gly (1:2), a significant increase (*p* < 0.01) was seen in fish injected intraperitoneally with saline compared to fish injected with 3000 µM Bet:Gly (1:2). Similar results were obtained between animals injected with 750 µM and 3000 µM DES (*p* < 0.01). Likewise, significant differences (*p* < 0.05) were also found between animals injected with 750 µM and 1500 µM. As for animals injected with the individual components, for example, betaine, a significant increase (*p* < 0.0001) was observed compared to fish injected with saline solution and the fish injected with 500 µM betaine. Similarly, significant differences (*p* < 0.01) were also detected, when comparing fish injected with 250 µM and 500 µM betaine. However, a significant reduction (*p* < 0.001) was seen when compared animals injected intraperitoneally with 500 µM and 1000 µM of betaine. Regarding activity of this enzyme in animals exposed to glycerol, a significant increase (*p* < 0.01) was observed comparing fish injected intraperitoneally with saline solution and fish injected with 1000 µM glycerol. The same differences (*p* < 0.05) were observed when comparing animals injected with 250 µM and 1000 µM glycerol.

#### Superoxide dismutase activity 

Figure 1D represents the average SOD activity determined in *D. rerio*. Regarding SOD activity, animals injected with the highest concentration of Bet:Gly (1:2) (3000 µM) showed a significant increase (*p* < 0.05) compared with animals injected with saline solution and those injected with 750 µM Bet:Gly (1:2), while animals injected with betaine showed a significant SOD increase (*p* < 0.001) between fish injected with 0 µM and fish injected with 250 µM betaine. However, a significant decrease (*p* < 0.001) was detected between fish injected intraperitoneally with a concentration of 250 µM and fish injected with a concentration of 1000 µM of betaine.

#### Total antioxidant capacity

TAC mean concentrations measured in *D. rerio* are shown in Fig. 1E. Regarding TAC results, a significantly increase (*p* < 0.01) was detected between animals injected with saline solution and animals injected with high concentration of Bet:Gly (1:2) (3000 µM). Moreover, the same was found between animals injected with 750 µM and 3000 µM and between animals injected with 1500 µM and 3000 µM Bet:Gly (1:2) (*p* < 0.05). Considering TAC concentration, a significant trend to increase (*p* < 0.05) was observed between animals intraperitoneally injected with saline solution and those injected with 250 µM betaine. However, a considerable TAC decrease (*p* < 0.05) was seen between animals injected intraperitoneally with 250 µM and 500 µM. Significant differences (*p* < 0.01) were also found between animals injected with 250 µM and the highest concentration tested (1000 µM) of betaine.

#### Lipid peroxidation assay

The average concentration of malonaldehyde (MDA) determined in *D. rerio* is shown in Fig. 1F. MDA levels increased significantly (*p* < 0.05) between animals injected with saline solution and animals injected with 750 µM and 1500 µM Bet:Gly (1:2). On the other hand, the opposite was observed between animals injected with 1500 µM and those injected with 3000 µM Bet:Gly (1:2), where a significant decrease (*p* < 0.01) was observed. Regarding the MDA concentrations in animals injected with betaine, a significant reduction (*p* < 0.05) was seen between animals injected with saline solution and those injected with 1000 µM betaine.

All correlations between the different enzymes are shown in supplementary material (Supplementary Table 1).

### Effects of Bet:Sor:W (1:1:3) upon intraperitoneal injection 

#### Mortality rate

The mortality rate registered throughout the assay was not significant (Table 4); however, a rate of 30 and 20% death was observed when animals were injected with a concentration of 1250 µM and 5000 µM, respectively. This mortality may be associated with perforation of the swim bladder during injection rather than the compound itself.

**Table 4:** Percentage of death of *Danio rerio* by concentration of Bet:Sor:W (1:1:3)

	**Bet:Sor:W (1:1:3)**
0 µM	0
1250 µM	30%
2500 µM	0
5000 µM	20%

#### Glutathione S-transferase

**Fig. 2:**
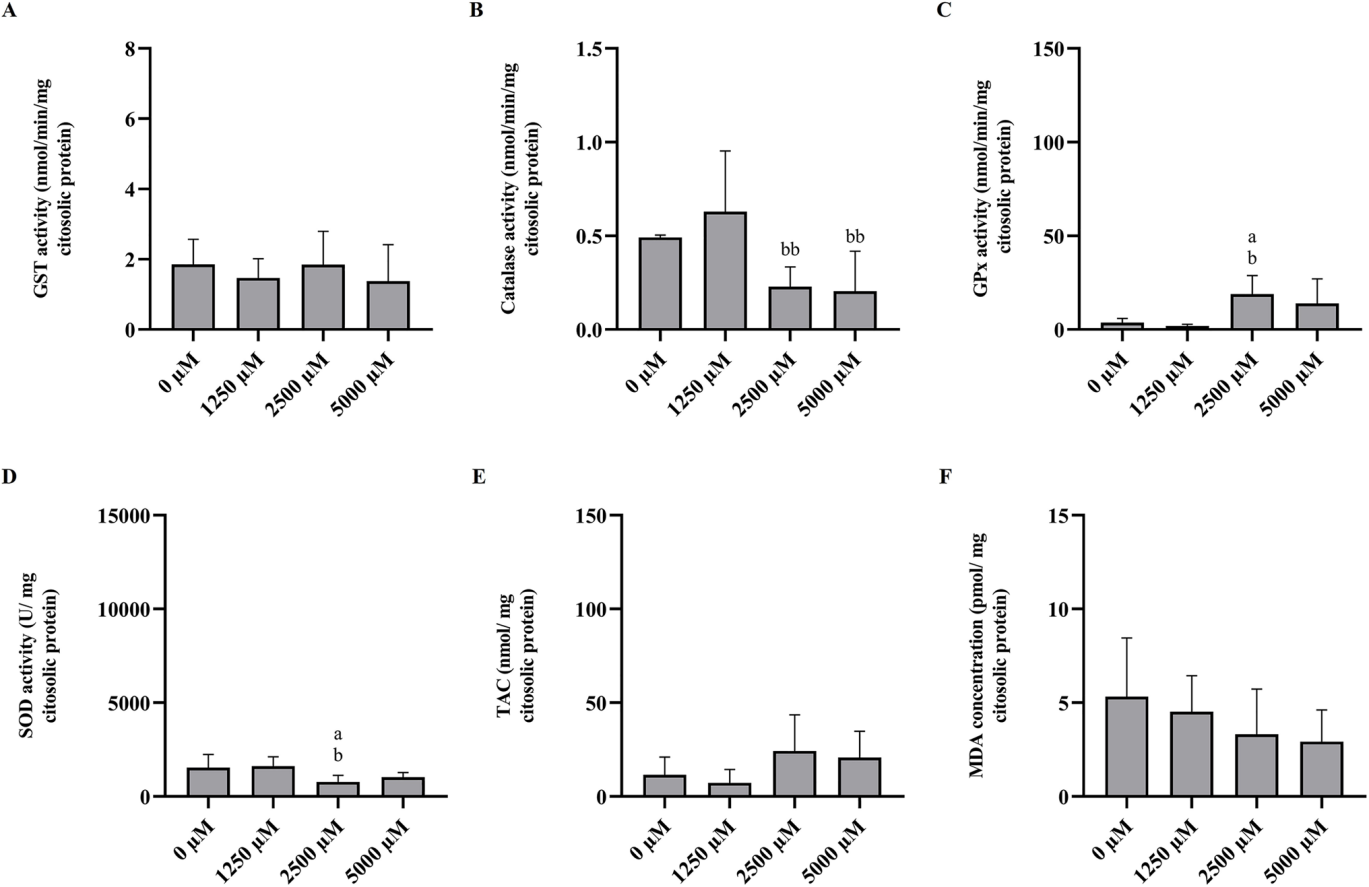
**A** GST activity, **B** CAT activity, **C** GP_x_ activity, **D** SOD activity, **E** TAC, and **F** MDA concentration after *D. rerio* injection with different concentrations of Bet:Sor:W (1:1:3). All data was represented as mean ± s.d.. a, significant differences in comparison to control; b, significant differences compared with 250 µM of Bet:Sor:W (1:1:3). One letter corresponds to significant differences with *p* < 0.05 and two repeated letters to *p* < 0.01

The GST activities mean concentrations measured in *D. rerio* are shown in Fig. 2A. However, although the highest GST activities were measured in fish injected with DES Bet:Sor:W (1:1:3), no discernible variations between the treatments or the controls were found.

#### Catalase

The CAT activity mean concentration measured in *D. rerio* are shown in Fig. 2B. Regarding catalase activity in animals exposed to Bet:Sor:W (1:1:3), a considerable reduction (*p* < 0.01) was detected between fish intraperitoneally injected with 1250 µM Bet:Sor:W (1:1:3) and with fish injected with 2500 µM and 5000 µM Bet:Sor:W (1:1:3).

#### Glutathione peroxidase activity

Figure 2C shows the average levels of GPx activity discovered in *D. rerio*. Regarding the activity of this enzyme in animals exposed to Bet:Sor:W (1:1:3), a considerable increase (*p* < 0.05) was observed between fish injected intraperitoneally with saline solution and fish injected with 2500 µM Bet:Sor:W (1:1:3). Similar results were obtained between animals injected with 1250 µM and 2500 µM Bet:Sor:W (1:1:3).

#### Superoxide dismutase activity

Figure 2D represents the mean SOD activity determined in *D. rerio*. A significant decrease (*p* < 0.05) was observed between fish injected with saline solution and fish injected with 2500 µM Bet:Sor:W (1:1:3). Similar results were observed between animals injected with 1250 µM and 2500 µM Bet:Sor:W (1:1:3).

#### Total antioxidant capacity (TAC)

The TAC mean concentrations determined in *D. rerio* are presented in Fig. 2E. Although higher TAC concentrations were measured in fish injected with Bet:Sor:W (1:1:3), no significant differences were found in comparison to controls or among treatments.

#### Lipid peroxidation assay

The MDA mean concentration measured in *D. rerio* are shown in Fig. 2F. Regarding MDA, no significant differences were found in comparison to controls or among treatments.

In addition, all correlations between the different enzymes are shown in supplementary material (Supplementary Table 2).

## Discussion

No significant mortality was noticed during the exposure period, indicating that the Bet:Gly (1:2) and Bet:Sor:W (1:1:3) systems, as well as the individual compounds betaine and glycerol, do not endanger fish survival, at least not at the concentrations that were examined during the trials (0, 3000 µM; 0, 5000 µM; and 0, 1000 µM, respectively, for the intraperitoneal injections). Animals receiving the same treatment showed some variation in their outcomes, which can be attributed to elements like gender or genetic predisposition (Gagnon and Hodson 2012).

In the present work, a small increase in oxidative stress enzymes (glutathione peroxidase activity) and total antioxidant capacity was observed suggesting that system Bet:Gly (1:2) does show low or no toxicity. With respect to Bet:Sor:W (1:1:3), it was observed a slight increase in GP_x_; however, overall results suggest that this DES shows low or no toxicity. A tendency to increase in GST activity was observed when considering animals injected with Bet:Gly (1:2) system; however, this increase is only significant at the highest concentration (3000 µM). This increase was associated with a significant positive correlation between GST and SOD (*r* = 0.4156) and between GST and GPx (*r* = 0.5654). This did not happen with individual components betaine and glycerol, wherein in the first case, an increase in the activity of that enzyme was observed in the lower concentration tested (250 µM), but at higher concentrations this was not significant. This may be explained due to described protective effects of betaine against oxidative stress (“Betaine Monograph,” 2003). Furthermore, it has been described in the literature as an increase in ROS production upon the administration of a concentration of 5 g/kg of glycerol in rats, which has also been shown to be responsible for renal injuries (Rieger et al. 2008). The concentration tested in this article is lower than the tested for the other articles, which can be one of the motifs for the different results.

There was a strong correlation between GP_x_ and TAC (*r* = 0.4839) in the animals injected with the systems Bet:Gly (1:2) and Bet:Sor:W (1:1:3). In the literature, it has been described that cells which had been treated with betaine:malic acid:proline or betaine:malic acid:glucose show that the antioxidant capacity increased with increasing DES concentrations after an oxygen radical antioxidant capacity (ORAC) assay (Radošević et al. (2018)). Similar results were obtained in the present work for the system Bet:Gly (1:2) at the highest concentrations tested (3000 µM). However, it must be noticed that these studies were performed with different biological models and different assays. The growth in antioxidant capacity can be considered a defense mechanism by the cells of living organisms, usually in response to the ROS increase. The increase in GP_x_ supports that activation of the antioxidant defense system is acting to protect cells as a result of ROS generation (Mowafy et al. 2021). Benlebna et al. (2018) used the system betaine:glycerol (1:2) with 10% of water (v/v) to extract phenolic compounds, rich in mono and diester, from green Arabic coffee beans (GCB). This extract was administered to rats at concentrations around 10 mg of chlorogenic acid equivalents per mL to understand the effects of administering this extract to rats for a short period of time. The main results obtained were the toxicity of this extract to rats, showed by increased mortality, excessive water consumption, dietary restriction, hepatomegaly, weight and adipose tissue loss, increased blood lipid levels, and plasma oxidative stress. On the other hand, Jesus et al. (2021) reported that similar DES (Bet:Gly:Suc:W (2:3:1:5) and Bet:Gly:T:W (2:3:1:5)) were less toxic for L929 cells at 10% than dimethyl sulfoxide, the cryopreservation agent which used in standard protocol for cryopreservation of mammalian cells. Likewise, Rodrigues et al. (2021) concluded that betaine/polyol-based DES (Bet:Gly (1:2), Bet:PG (1:3). and Bet:EG (1:3)) had low toxicity toward Caco-2 cell line, bacteria gram-positive and gram-negative, and wheat plant seeds. However, phytotoxicity studies conducted by the same authors showed that Bet:Gly (1: 2) and Bet:PG (1:3) inhibited more than 50% of the shoots elevation at the highest tested concentration.

In general, betaine is able to inhibit the activity of most enzymes. At higher concentrations of betaine (1000 µM), the activity of most enzymes is lower, when compared to lower concentrations of betaine (250 µM) injected in fish. Hence, for GST and SOD, there are significant differences between the control and the lowest concentration (250 µM) of betaine. Catalase is the only enzyme that does not follow this trend. The increase in SOD activity, not followed by the increase in catalase, can be attributed to other antioxidant enzymes (e.g. glutathione peroxidase, peroxiredoxin) acting to scavenge formed, for example, H_2_O_2_. Another possible justification for this biomarkers response is related to the capacity of betaine to protect cells against stress (“Betaine Monograph,” 2003).

Regarding glycerol, it was observed a significant increase in GP_x_ activity. The significant increase in GP_x_ activity was associated with a tendency to decrease in catalase activity, probably because GP_x_ and catalase are two enzymes that catalyze similar reactions, which is the conversion of hydrogen peroxide into water and molecular oxygen (Pandey and Rizvi 2010).

In animals injected with the Bet:Sor:W (1:1:3) system, it was observed a decrease in GP_x_ (when comparing animals injected with control and those injected with 2500 µM DES), which might be a sign that the antioxidant defense system is not working properly. It was observed a low negative correlation, in the Bet:Sor:W (1:1:3) system, between CAT and GP_x_ which can be justified by the point that these two enzymes are responsible for catalyzing the reduction of hydrogen peroxide (H_2_O_2_) into water and oxygen (O_2_) (Pandey and Rizvi 2010). A high positive correlation was observed between catalase and superoxide dismutase, which can be explained by the fact that the hydrogen peroxide that is converted by catalase into oxygen and water comes from a first stage, the SOD-catalyzed conversion of oxygen radicals to hydrogen peroxide (Pandey and Rizvi 2010). This correlation may be a reason for the significant decrease in SOD, despite the significant increase in GP_x_ activity. The decrease in SOD activity is similar to what was discussed by Rodrigues et al. (2021) that analyzed SOD activity levels in seedling-harvested leaves exposed to a concentration of 20 mg/mL of Bet:Gly (1:2), to a concentration of 5 or 20 mg/mL of Bet:EG (1:3) and to a mass concentration of 5 mg/mL of Bet:PG (1:3).

The tendency for MDA to decrease can be derived from the protective effect of GP_x_, which protect cells from injury.

By comparing the two different DES tested in this work, Bet:Gly (1:2) and Bet:Sor:W (1:1:3), no significant differences in toxicity were detected, suggesting low or no toxicity. These results were very similar to those found in the literature on different betaine based- DES tested in cell lines models. Some examples of these studies were Bet:Gly:Suc:W (2:3:15), Bet:T:W (4:1:10), Bet:Suc:Pro:W (5:2:2:1), and Bet:Xyl:W (2:1:3:1), reported by Jesus et al. (2021), that showed that L929 cells tolerate very high concentrations of these DES. Another study, by Radošević et al. (2018), reports that betaine:glucose (5:2), betaine:malic acid:glucose (1:1:1), and betaine:malic acid:proline (1:1:1) are highly tolerated by different cell lines (HeLa cells, HEK293T cells and MCF-7 cells). Additionally, Rodrigues et al. (2021) report that Bet:Gly (1:2), Bet:Gly:W (1:2:1.2), Bet:Gly:W (1:2:5.2), Bet:Gly:W (1:2:10.2), Bet:PG (1:3), Bet:PG:W (1:3:1.6), Bet:PG:W (1:3:5.6), Bet:PG:W (1:3:10.6), Bet:EG (1:3), Bet:EG (1:3:1.2), Bet:EG (1:3:5.2), and Bet:EG (1:3:10.2) show low cytotoxicity to Caco-2 cell line.

Therefore, in general, the in vivo studies of these DES and their individual components show low or no toxicity to zebrafish at the concentrations tested.

## Conclusions

The purpose of this study was to examine, for the first time, the in vivo toxicity of two different systems based on betaine, namely, Bet:Gly (1:2) and Bet:Sor:W (1:1:3), and its individual components, betaine and glycerol. This was performed by exposing zebrafish via intraperitoneal injection. The results obtained are highly relevant towards the understanding of DES and given that they offer new information about the effects of the two DES in fish. The results suggest that the systems Bet:Gly (1:2) and Bet:Sor:W (1:1:3) tested do not demonstrate relevant toxicity up to a concentration of 3000 µM and 5000 µM, respectively, despite changes in some enzymatic activities. However, the alterations in enzymatic activities were not demonstrated by lipid peroxidation results, which can be the result of increased reactive oxygen species. Similarly, this was also observed with the individual components tested, indicating with some certainty the possibility of its use in several industries when used up to the concentrations tested in this work. The results obtained suggest that these two DES have the ability to be employed as a new class of solvents of respect the green principles that can be used in several applications, for example, pharmaceutical and cosmetic industry and cell cultures without harming living organisms.

## Supplementary Information

Below is the link to the electronic supplementary material.

Supplementary file1 (DOCX 16 KB)

## Data Availability

Data will made available on request.
