# Supplementary material for: Uncovering biodegradability and biocompatibility of betaine-based deep eutectic systems
Source: Environ Sci Pollut Res Int. 2023 Jan 6;30(14):40218–29. doi: 10.1007/s11356-022-25000-6 (PMC10067644; doi:10.1007/s11356-022-25000-6)
Supplement: Supplementary file 1 — Supplementary file1 (DOCX 16 KB) [file 11356_2022_25000_MOESM1_ESM.docx]

|  | **LPO** | | | **TAC** | | | **SOD** | | | **GP_X_** | | | **CAT** | | |
| --- | --- | --- | --- | --- | --- | --- | --- | --- | --- | --- | --- | --- | --- | --- | --- |
|  | Bet: Gly  (1:2) | Bet | Gly | Bet: Gly  (1:2) | Bet | Gly | Bet: Gly  (1:2) | Bet | Gly | Bet: Gly  (1:2) | Bet | Gly | Bet: Gly  (1:2) | Bet | Gly |
| **GST** | ----- | ----- | ----- | ----- | 0.5096  p <0.01 | ----- | 0.4156  p <0.05 | 0.5301  p <0.01 | 0.3492  p <0.05 | 0.5654  p <0.001 | ----- | ----- | ----- | ----- | ----- |
| **CAT** | ----- | ----- | ----- | ----- | - 0.3524  p <0.05 | ----- | ----- | - 0.4161  p <0.05 | ----- | ----- | ----- | ----- |  |  |  |
| **GP_X_** | ----- | ----- | ----- | 0.4839  p < 0.01 | ----- | ----- | 0.5828  p <0.001 | ----- | ----- |  |  |  |  |  |  |
| **SOD** | ----- | ----- | 0.3524  p <0.05 | 0. 4874  p <0.01 | 0.7327  p<0.0001 | 0.3742  p <0.05 |  |  |  |  |  |  |  |  |  |
| **TAC** | ----- | ----- | ----- |  |  |  |  |  |  |  |  |  |  |  |  |

***Supplementary table 1*:** correlations between the different enzymes involved in antioxidant process (GST, CAT, GP_x_ and SOD), TAC and Lipid peroxidation in animals exposed to Bet:Gly (1:2), Betaine and Glycerol.

|  | **LPO** | **TAC** | **SOD** | **GP_X_** | **CAT** |
| --- | --- | --- | --- | --- | --- |
| **GST** | ----- | ----- | ----- | ----- | ----- |
| **CAT** | ----- | ----- | 0.5171  p < 0.01 | - 0.4933  p < 0.05 |  |
| **GP_X_** | ----- | 0.5692  p < 0.01 | ----- |  |  |
| **SOD** | ----- | ----- |  |  |  |
| **TAC** | ----- |  |  |  |  |

***Supplementary table 2*:** correlations between the different enzymes involved in antioxidant process (GST, CAT, GP_x_ and SOD), TAC and Lipid peroxidation in animals exposed to Bet:Sor:W (1:1:3)
